# Surgical management of ascending aortic pseudoaneurysm in a 2-year-old boy: a case report

**DOI:** 10.1186/s13256-018-1625-z

**Published:** 2018-04-17

**Authors:** Merna Atiyah, Shazia Mohsin, Lama Al Faraidi, Khaled Al-Hawri, Abdulmajeed Al Otay, Khalid Al Najashi

**Affiliations:** 1 Pediatric Cardiology Department, Prince Sultan Cardiac Center, P.O. Box 99911, Postal Code 11159 Riyadh, Kingdom of Saudi Arabia; 2 Pediatric Cardiac Surgery Department, Prince Sultan Cardiac Center, Riyadh, Kingdom of Saudi Arabia

**Keywords:** Ascending aortic pseudo-aneurysm, Congenital heart surgery, Coarctation repair, Case report

## Abstract

**Background:**

Aortic pseudoaneurysms are rare but life-threatening complications usually seen after cardiac surgery. The causes could be multifactorial such as infection or trauma.

**Case presentation:**

We report the surgical management of a postoperative pseudoaneurysm of the ascending aorta caused by methicillin-resistant *Staphylococcus aureus* in a 2-year-old Middle Eastern boy who had undergone ventricular septal defect closure, subaortic membrane resection, and pulmonary artery de-banding. He was immediately operated on for resection of the aneurysm. A computed tomography scan at 2 months following surgery showed no aneurysm. Antibiotics were continued for 6 weeks and our patient was discharged with negative blood cultures.

**Conclusion:**

Early diagnosis and appropriate treatment of such rare complication can be lifesaving.

## Background

Aortic pseudoaneurysms are rare, life-threatening sequelae of cardiac surgery [1]. They can also occur as a result of infections, genetic disorders, or trauma [2–5]. They are uncommon in children. Several cases have been reported, most of which were secondary to bacterial endocarditis [6–8]. Our case highlights a rare postoperative complication that has not been reported previously in our region. Various management strategies have been suggested, including surgical and catheter-based interventions. Because of the age and size of our patient, and the infected nature of the lesion, we opted for surgical intervention. Timely diagnosis with effective surgical treatment in combination with antibiotic coverage can result in good survival without complications in such life-threatening situations.

## Case presentation

**Fig. 1:**
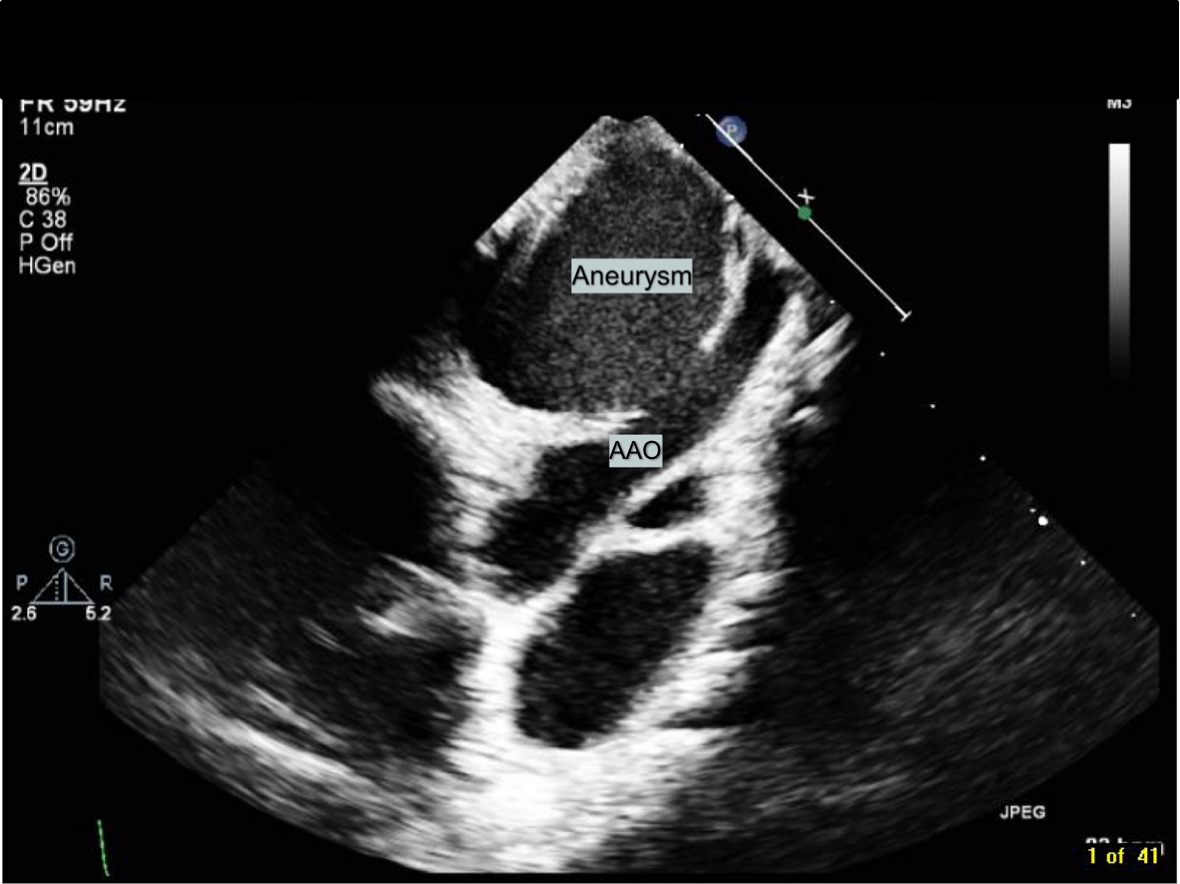
Transthoracic echocardiography, suprasternal view, revealing a huge pseudoaneurysm arising from the anterior wall of the ascending aorta. *AAO* ascending aorta

**Fig. 2:**
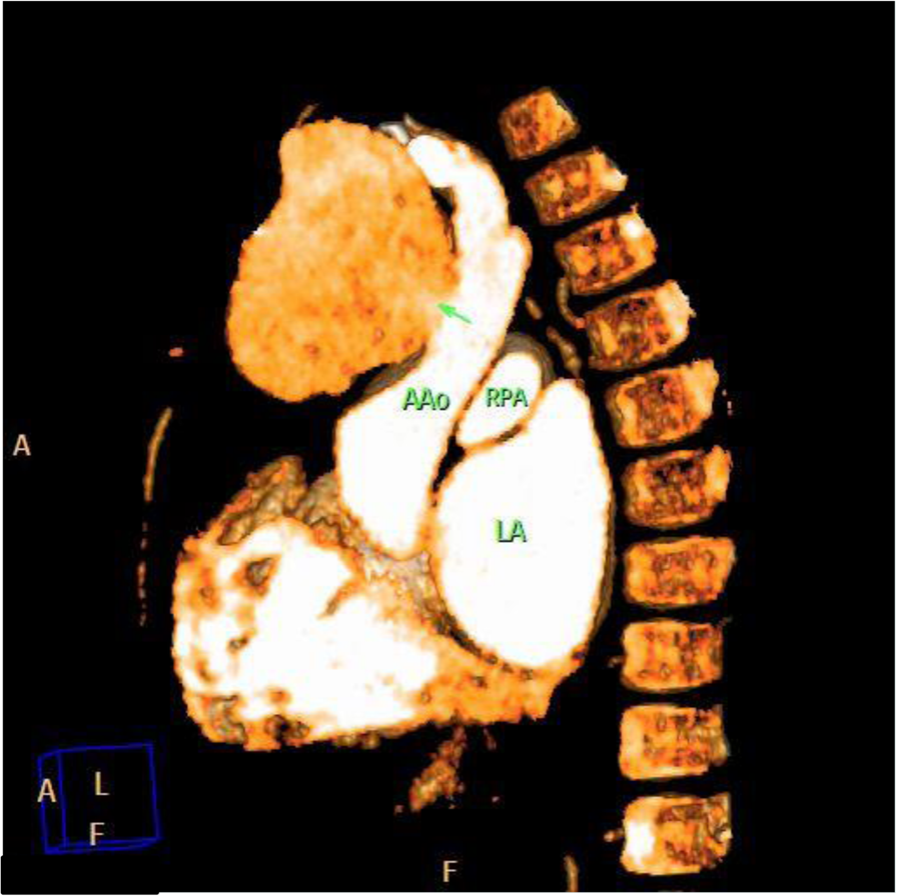
Computed tomography angiogram (colored) which shows a pseudoaneurysm arising from the anterior wall of the ascending aorta (arrow). Dimensions were 4.5 × 4.0 × 3.5 cm with a 10–11 mm neck. The aneurysm extended to the right and superiorly causing rightward deviation of the superior vena cava and innominate vein. *AAo* ascending aorta, *LA* left atrium, *RPA* right pulmonary artery

**Fig. 3:**
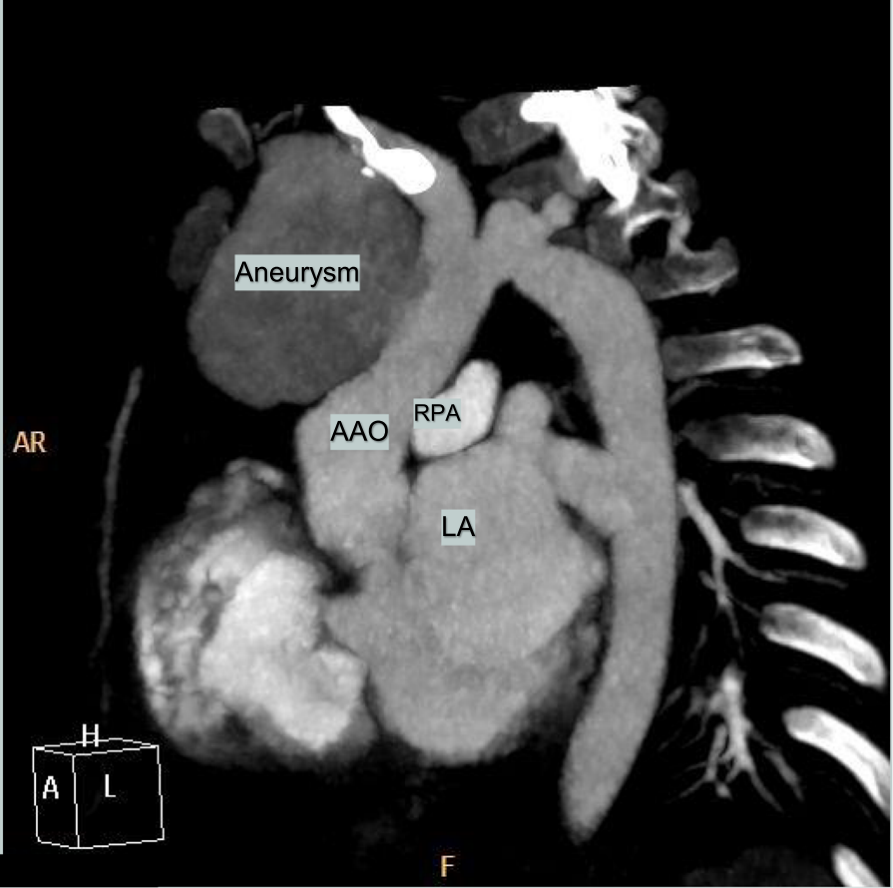
Computed tomography angiogram which shows a pseudoaneurysm arising from the anterior wall of the ascending aorta. Dimensions were 4.5 × 4.0 × 3.5 cm with a 10–11 mm neck. The aneurysm extended to the right and superiorly causing rightward deviation of the superior vena cava and innominate vein *AAO* ascending aorta, *LA* left atrium, *RPA* right pulmonary artery

Our patient is a 2-year-old Middle Eastern boy, weighing 12 kg below fifth centile, height 88 cm at the tenth centile, with bicuspid aortic valve (BAV) coarctation of the aorta, a large inlet ventricular septal defect (VSD) and subaortic membrane with mild left ventricular outflow tract obstruction. He was the product of a consanguineous marriage and there is no history of congenital heart disease in the family. He underwent aortic arch repair and pulmonary artery (PA) banding at 10 days of life. After that he was on regular follow up at our center. He was started on antifailure medication and was referred for complete repair of VSD closure and PA debanding as he was not gaining weight. He was developmentally normal and no other noncardiac comorbidity was identified. The surgery was performed successfully and postoperative transesophageal echocardiography (TEE) showed no residual lesions. On day 5 postoperatively, he developed lethargy, fever, and sternal wound discharge. He had a temperature of 39 °C and tachycardia with heart rate of 140 beats per minute. He showed leukocytosis with a total white blood cell count of 16 × 109/L and raised acute phase reactants. Our patient had methicillin-resistant *Staphylococcus aureus* (MRSA) growth confirmed by two blood and wound cultures which showed sensitivity to vancomycin and meropenem. He was treated with intravenously administered antibiotics (meropenem and vancomycin). The rest of laboratory parameters including renal, liver, and urine analyses were normal. Repeat blood cultures at 3 weeks of treatment were still positive for MRSA. Rifampicin was added. His follow-up echocardiography (Fig. 1) at 3-weeks postoperatively, while still on antibiotics, revealed a large pseudoaneurysm arising from the anterior wall of his ascending aorta (AsAo). “Smoky” blood flow into the aneurysm cavity was seen, but no thrombi or vegetation was detected. These findings were confirmed on computed tomography (CT) angiogram, which showed a pseudoaneurysm arising from the anterior wall of the AsAo. The lesion was 4.5 × 4.0 × 3.5 cm in size and had a 10–11 mm neck. It extended to the right and superiorly, causing rightward deviation of his superior vena cava and innominate vein (Figs. 2 and 3). He was immediately sent to the operating theater. Surface cooling was started early while exposing his right femoral vessels. A 3.5 mm Gore-Tex tube was anastomosed to the side of his proximal right common femoral artery using a 7/0 polypropylene stitch. After heparin was given, a 10 Fr arterial cannula was inserted into the Gore-Tex tube, and was fixed. When his core temperature reached 28 °C, the lower part of his sternum was opened and his right atrium was cannulated with a 24 Fr metal-tip cannula. A cardiopulmonary bypass was started with a full flow of 120 ml/kg, and cooling was continued on bypass until a core temperature of 20 °C was reached. His head was additionally cooled with ice packs. The dissection was then continued. When the upper part of his sternum was reached, the sac of the false aneurysm was opened. His heart was fibrillating at this point. Very low flow was used to improve exposure. A 1 × 1 cm hole at the site of the previous aortic cannulation was found. The edges of the hole and the surrounding tissue appeared infected and friable. Cultures were taken. A cross-clamp was then applied to his AsAo distal to the hole. No cardioplegia was used. A bovine pericardial patch was used to repair the ascending aortic wall. Because of tissue friability, the patch was sutured away from the edges using 7/0 polypropylene mattress continuous stitches. This was further reinforced by an outer 6/0 polypropylene stitch. BioGlue was applied over the patch. After rewarming, the cardiopulmonary bypass was weaned off easily. The femoral arterial line was removed and the Gore-Tex tube was cut short and clipped. The cross-clamp time was 28 minutes, and bypass time was 111 minutes.

**Fig. 4:**
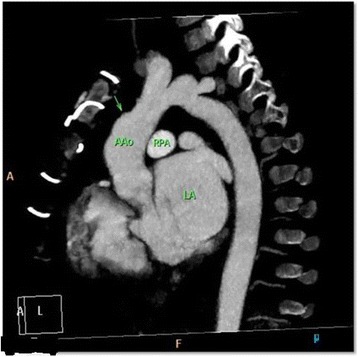
Computed tomography angiogram after surgery showed no aneurysm (arrow). *AAo* ascending aorta, *LA* left atrium, *RPA* right pulmonary artery

Our patient had a smooth postoperative course with no signs of infection and no neurological or ischemic complications. Antibiotics were continued for 6 weeks and he was discharged with negative blood cultures and normal inflammatory markers. We followed our patient 6 weekly for the first 3 months, then every 6 months, then annually in clinic and repeated a cardiac CT scan (Fig. 4) at 2 months following surgery which showed no aneurysm. At 6-month out-patient follow up he had an unremarkable clinical examination and an echocardiogram showed no signs of aneurysm and had normal function. He gained weight to 15 kg.

## Discussion

Acquired aneurysms of the AsAo, although rarely seen, are most commonly found at cannulation sites and suture lines, particularly after repair of coarctation of the aorta [9]. As reported these aneurysms are diagnosed with a high index of suspicion as symptoms are usually absent or nonspecific. They carry a high risk of mortality if diagnosed late and surgery is performed after the rupture [6]. In the past, infected aneurysms were seen mostly as a result of infected endocarditis and repaired valvular diseases. These aneurysms can occur as direct invasion of aortic intima by circulating bacteria or through lymphatics in the presence of infection and mediastinitis. An added risk is therapeutic intervention like aortic cannulation [6]. Similarly, our patient showed signs of infection on postoperative day 5 and his echocardiogram revealed a large aneurysm at the site of his aortic cannulation which was confirmed by CT angiogram. Our patient had MRSA growth confirmed by two blood and wound cultures, which along with other streptococcal species is one of the common organism isolated after mycotic aneurysm [10]. It is extremely important to promptly diagnose these cases with a high index of suspicion especially after coarctation repair or history of aortic cannulation like in our case. An echocardiogram is a noninvasive, immediately available screening tool; cardiac CT angiogram is more sensitive. In our patient, with a high index of suspicion due to wound infection, an aortic cannulation and echocardiogram were done immediately and, later, cardiac CT was done to confirm the diagnosis.

Both immediate and long-term types of pseudoaneurysms have been reported [7]. Various treatments of pseudoaneurysm are advocated in the literature. There is no well-defined treatment for this lesion in the pediatric population. Both percutaneous and surgical repairs have been advocated in the literature. However, percutaneous closure has been successfully reported in the adult population only [11, 12]. Surgical repair still remains the gold standard, especially because of the infected nature of the lesion that precludes the use of percutaneous devices. The main challenge for the surgeon is to plan an approach to the lesion without risking hemodynamic compromise, neurological insults that might be caused by air embolism, and hypotension secondary to bleeding or heart manipulation during dissection. Adhesions from previous cardiac surgery increase these risks. Various surgical approaches have been employed, such as femoral or neck cannulation to establish cardiopulmonary bypass, deep hypothermic circulatory arrest, and antegrade cerebral perfusion [4]. Barth *et al*. in their case report of a similar case used the left common iliac artery for repair of a pseudoaneurysm of the AsAo in a 16-month-old girl [7].

Our patient had a tear at the site of aortic cannulation that appeared to be caused by bacterial infection with MRSA. Our approach included surface cooling, femoral cannulation mitigated by the use of a Gore-Tex graft for arterial cannulation, right atrial cannulation, and use of deep hypothermia. We found this method to be a safe and reproducible. A similar case was reported by Miyaji *et al*., where the common iliac artery was cannulated. Their case was postoperative mycotic pseudoaneurysm in the AsAo secondary to mediastinitis caused by MRSA after a modified Fontan procedure [13].

## Conclusions

In conclusion, aortic pseudoaneurysms are rare but recognized complications of cardiac surgery that can be life-threatening. Prompt diagnosis and management, as in our case, can result in successful outcomes.
